# Estimated six per cent loss of genetic variation in wild populations since the industrial revolution

**DOI:** 10.1111/eva.12810

**Published:** 2019-06-05

**Authors:** Deborah M. Leigh, Andrew P. Hendry, Ella Vázquez‐Domínguez, Vicki L. Friesen

**Affiliations:** ^1^ Department of Biology Queen's University Kingston Ontario Canada; ^2^ WSL Swiss Federal Research Institute Birmensdorf Switzerland; ^3^ Department of Biology McGill University Montréal Quebec Canada; ^4^ Redpath Museum, McGill University Montréal Quebec Canada; ^5^ Departamento de Ecología de la Biodiversidad, Instituto de Ecología Universidad Nacional Autónoma de México Ciudad de México Mexico

**Keywords:** genetic erosion, genetic variation, temporal diversity

## Abstract

Genetic variation is fundamental to population fitness and adaptation to environmental change. Human activities are driving declines in many wild populations and could have similar effects on genetic variation. Despite the importance of estimating such declines, no global estimate of the magnitude of ongoing genetic variation loss has been conducted across species. By combining studies that quantified recent changes in genetic variation across a mean of 27 generations for 91 species, we conservatively estimate a 5.4%–6.5% decline in within‐population genetic diversity of wild organisms since the industrial revolution. This loss has been most severe for island species, which show a 27.6% average decline. We identified taxonomic and geographical gaps in temporal studies that must be urgently addressed. Our results are consistent with single time‐point meta‐analyses, which indicated that genetic variation is likely declining. However, our results represent the first confirmation of a global decline and provide an estimate of the magnitude of the genetic variation lost from wild populations.

## 1 INTRODUCTION

Genetic variation underpins population fitness and adaptive potential (Hoffmann, Sgrò, & Kristensen, 2017; Reed & Frankham, 2003). As a consequence, it is a key component of species extinction risk, particularly under global climate change. Genetic variation can be lost in a single generation, but its replenishment may take hundreds of generations (Nei, Maruyama, & Chakraborty, 1975). Safeguarding genetic variation is therefore considered fundamental to mitigating biodiversity loss, and the maintenance of genetic variation is an Aichi Target for 2020 (CBD, 2011). Furthermore, genetic variation is classed as an Essential Biodiversity Variable, meaning that its status should be monitored to help prevent harmful biodiversity loss (Pereira et al., 2013 *)*. Genetic variation is also a component of the planetary boundaries that humanity must operate within for our own survival (Mace et al., 2014). As human activities are driving declines in many wild populations (WWF, 2016), genetic variation is also likely declining (Frankham, 1996). Despite its importance, the magnitude of ongoing genetic variation loss has not been assessed. This is essential because variation loss cannot easily be inferred. Indirect estimates based on changes in population size or extinction rates will likely be inaccurate (e.g. Steffen et al., 2015). Processes like hybridization can also increase genetic variation (e.g. Grossen, Keller, Biebach, & Croll, 2014), making the overall trends unclear in some species.

Single time‐point analyses have shown that mitochondrial sequence diversity is substantially lower in geographical regions heavily affected by human activity (Miraldo et al., 2016). Nuclear variation is also lower in fragmented populations relative to undisturbed populations (DiBattista, 2008), suggesting that genetic variation is likely in global decline due to a continued increase in habitat fragmentation (Ellis, Goldewijk, Siebert, Lightman, & Ramankutty, 2010). Nonetheless, single time‐point analysis cannot disentangle contemporary declines in variation from historically low values caused, for example, by ancient population crashes, colonization population dynamics, or species‐specific traits and histories (Díez‐del‐Molino, Sánchez‐Barreiro, Barnes, Gilbert, & Dalén, 2018; Habel, Husemann, Finger, Danley, & Zachos, 2014). Assessment of global changes in within‐population genetic variation requires cross‐generational genetic comparisons of the same population, ideally over long periods of time. Many such cross‐generational studies have been performed, yet the resulting data have not been synthesized into a global estimate of the direction and magnitude of changes in genetic variation. Here, we analysed temporally repeated measures of population genetic variation that were at least one organismal generation apart. We compared estimates of genetic variation, namely heterozygosity and allelic diversity because these were consistently reported across time points, to assess the magnitude of genetic variation decline. We also examined factors associated with the direction and magnitude of changes observed.

## 2 METHODS

We searched Google Scholar (20/02/2018–24/05/2018 and 25/02/2019) for peer‐reviewed publications that repeatedly sampled nuclear variation in the same population. Keywords used were "museum sequencing," "genetic diversity time," "ancient DNA," "temporal diversity genetics," "historic specimens," "museum specimens," "museum specimens genomics," "temporal genetic dynamics," and "ghost alleles." We examined 5970 peer‐reviewed publications and identified 88 publications on 91 species that met our criteria. Five species were independently studied multiple times (*n* = 13), meaning that 99 independent temporal estimates of genetic variation were available. Metrics of genetic variation, including expected heterozygosity (defined as the proportion of heterozygotes expected under Hardy–Weinberg equilibrium given the allele frequencies) (Allendorf, Luikart, & Aitken, 2013), observed heterozygosity and allelic diversity (as measured by allelic richness, number of alleles, nucleotide diversity [π] or method‐of‐moments inbreeding [*F*_H_]), were collected from each species within a study (i.e. each independent estimate). Eighty‐eight estimates were based on microsatellite markers, nine on single nucleotide polymorphisms, and two on restriction enzyme‐based markers. Studies spanned an average of 97 years (±439 *SD*) or 27 generations (±44 *SD*). Eighty‐four estimates spanned more than one generation.

We recorded the mean genetic variation metrics for two time point, henceforth “historic” and “modern,” from each species within a study. Historical time points, defined as the age of the oldest samples, were made possible through archival or ancient DNA specimens. Many of the oldest historical samples were collected towards the end of the industrial revolution (~1840), though the earliest sample was from 2.3 kY BC. The average historical sample was from 1908. Modern samples were defined as the youngest samples, and the average modern sample was from 2005. Though multiple time points would be ideal for pinpointing when change in genetic variation began, many publications had only two time points and we were forced to limit the analysis to two time points. For each species within a study, we took an average of each metric of genetic variation per time point. Consequently even if diversity metrics were measured at the population level, we use only an average across all populations. This was necessary because many studies only report historical genetic variation in this way. To maintain consistency, only populations that were sampled in both the historic and modern time points were included in our average. An exception was made if a population from the historic time point went extinct before modern samples were taken. Such extinct populations were included in the historic time‐point average only. For each species in a study, we recorded the length of time separating the oldest and newest sample in generations, as well as the start date of the study. If multiple time points were reported, we took the two furthest apart. If multiple estimates of generation time were given for a species, we used the median. If no generation time was given in the study reporting the genetic estimates, we used estimates for the same species from peer‐reviewed literature. To account for potential research bias towards bottlenecked or inbred species, which would inflate estimates of diversity loss, we recorded whether the paper discussed if the time series began before or after a major population crash or “bottleneck” (hereafter bottleneck status), and *IUCN Red List of Threatened Species* status. When the *IUCN* ranking contradicted the study's description, we deferred to the status in the paper. The country and continent of the study were also recorded, as well as whether the focal populations were from islands or continental mainlands. To check for a bias in taxonomic representation and examine whether declines were unequal across taxa, we recorded the taxonomic class of each species. We also recorded the mean census population size at each time point, as well as the minimum number of genetic markers used. However, because population size estimates were reported in only 36 studies, this explanatory variable was excluded from our analysis. Furthermore, because genetic variation is more closely related to effective population size and not census size (Charlesworth, 2009), census size is unlikely to have an association with genetic variation. Unfortunately, effective population sizes were impossible to include due to huge confidence intervals on estimates or the use of multiple estimation methods, each giving a very different value. We also recorded sample size, which was fairly even across time points (116 ± 153.4 *SD* in historical time points and 135 ± 146.5 *SD* in modern). Finally, we also recorded if any markers in the historic and modern time points deviated from Hardy–Weinberg equilibrium proportions.

### 2.1 Global trends in genetic variation

We used three separate paired *t* tests to examine whether the mean observed heterozygosity, expected heterozygosity, and allelic diversity differed between time points. Pairs consisted of the modern and historic time‐point values of allelic diversity, expected heterozygosity or observed heterozygosity for each species in each independent study that spanned more than one generation. Thus, species that were studied multiple times were initially considered as independent pairs. Paired analyses were necessary because they preserved the vital connection between the time points from the same study. This ensured that we assessed changes in diversity using the unique historical baseline for each modern estimate. A paired analysis is also technically necessary because each marker set will suffer from different ascertainment biases and study‐specific limitations. Furthermore, paired data points are not independent and should not be separated. To meet the assumption of normality, we log‐transformed the values for each time point (results were stable without transformation). Due to multiple allelic diversity metrics, it was also necessary to standardize the values of allelic diversity by dividing both the historic and modern values by the historic value. The difference in variance that causes between historic and modern values does not violate the assumptions of the paired *t* test, because it is the difference between the paired values that the test assumptions apply to (McDonald, 2014). The standardization was not necessary for heterozygosity estimates because they were always reported as expected or observed heterozygosity. Five species were present in multiple studies (*n* = 13). These values were not initially excluded and were treated independently because the studies are independent and the populations were different evolutionary significant units. However, to control for potential bias due to unequal taxonomic representation, we averaged across each taxonomic class and repeated the paired *t* tests on this reduced data set.

### 2.2 Factors affecting loss of genetic variation

To examine factors influencing the total change between time points in expected heterozygosity and allelic diversity, we used linear mixed‐effects models (lme4, version 1.1.18; Bates, Maechler, Bolker, & Walker, 2015) and backwards stepwise deletion. Observed heterozygosity was not examined because it did not change significantly between time points. To account for unequal taxonomic representation, taxonomic class was included as a random effect. The bottleneck status, age of the oldest sample, if the focal populations were on the island or continental mainland, and number of generations between time points were included as explanatory variables in the full model. The full model contained all listed variables and no interactions because balanced replication was not present across the factors. The response variables were scaled (i.e. the total change in each metric was divided by the historical value), because relative loss is biologically important and this ensured the response variables met the model assumptions. The linear mixed‐effects model examining change in allelic diversity was constrained to allelic richness. This is because the different allelic diversity metrics cannot be combined in this analysis, as they are highly variable when combined. Model selection was performed by removing the variable with lowest *t* value from each nested model, followed by confirming the variable's nonsignificance using a likelihood ratio test (chi‐squared). This was repeated until the final model contained only terms that resulted in significant change in the deviance of the two nested models (i.e. *p* < 0.05 and a *t* value > 2). Both models only examined studies spanning more than one generation. To ensure the data frame did not change with variable removal, species without a clear *Red List* status or bottleneck status were removed from the data set. Values of percentage change reported from the linear mixed‐effects models are for this reduced data set (*n* = 64, expected heterozygosity; *n* = 43, allelic richness).

A chi‐squared test was conducted to test whether deviation from Hardy–Weinberg equilibrium proportions was more common in historic or modern time points. This was to ensure the trends that we identified were not driven by inflated historical estimates of diversity due to false alleles from error‐prone historic samples (Wandeler, Hoeck, & Keller, 2007). We considered a deviation from Hardy–Weinberg equilibrium to have occurred if any marker proportions were significantly in disequilibrium after corrections for multiple testing. We also required that the tests were separated by time point. If the tests were conducted on data combined across time points, the study was scored as “NA.” The total number of marker proportions not in equilibrium was not considered, as this was typically low (1–2 markers).

All analyses listed above were conducted in R (v.3.5.0; R Core Team, 2018). All tests were two‐sided.

## 3 RESULTS

### 3.1 Global trends in genetic variation

We documented an average 5.4% (±18.8% *SD*) global decrease in mean expected heterozygosity (*t* = 2.74, mean of historical time point (log) = −0.590, mean of modern time point (log) = −0.672, mean of differences = 0.081, 95% confidence interval (CI) = 0.022–0.141, *df* = 69, *p* = 0.008, paired *t* test). A slightly higher decrease of 6.5% (±17.8% *SD*) was seen for mean allelic diversity (*t* = 3.46, mean of historical time point (log) = 0, mean of modern time point (log) = −0.094, mean of differences = 0.094, CI = 0.040–0.148, *df* = 72, *p* = 0.0009, paired *t* test). In contrast to expected heterozygosity and allelic richness, observed heterozygosity did not change significantly (nonsignificant increase of 1% ±19% *SD*; *t* = 0.42, mean of historical time point (log) = −0.7, mean of modern time point (log) = −0.713, mean of differences = 0.013, CI = −0.047–0.072, *df* = 60, *p* = 0.67, paired *t* test). Across taxonomic averages, we also identified a significant decline in expected heterozygosity of 3.8% (±4.9% *SD*; *t* = 2.47, mean of historical time point (log) = −0.560, mean of modern time point (log) −0.620, mean of differences = 0.04, CI = 0.0033–0.0771, *df* = 9, *p* = 0.0358, paired *t* test) and decline in allelic diversity of 3.7% (±10.1% *SD*). However, this was nonsignificant (*t* = 1.36, mean of historical time point (log) = 0, mean of modern time point (log) = −0.05, mean of differences = 0.049, CI = −0.0342–0.1340, *df* = 8, *p* = 0.2084, paired *t* test). Trends in observed heterozygosity remained nonsignificant (*t *= −1.51, mean of historical time point (log) = −0.664, mean of modern time point (log) = −0.649, mean of differences = −0.015, CI = −0.0375–0.0078, *df* = 8, *p* = 0.1686).

### 3.2 Factors affecting loss of genetic variation

**Figure 1:**
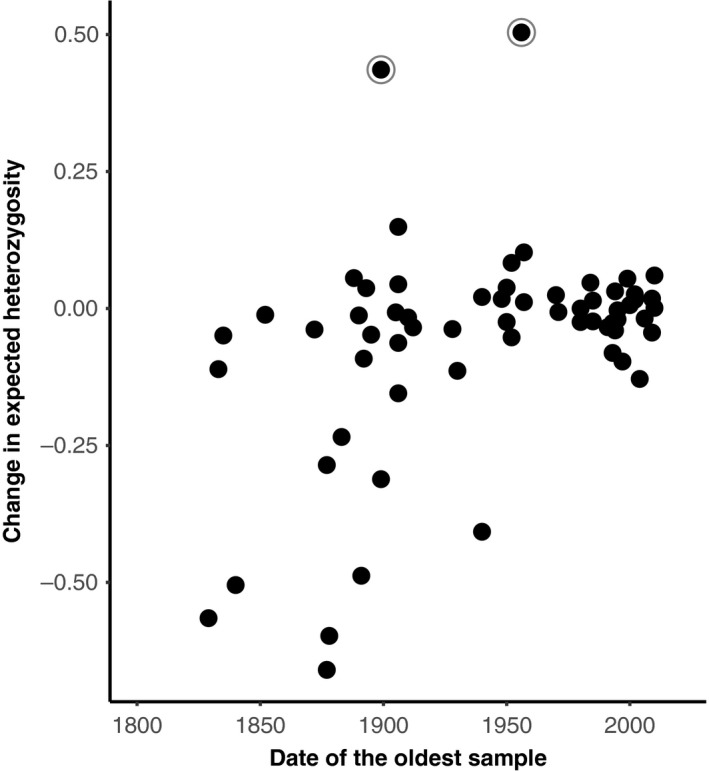
Proportional change in expected heterozygosity in modern time points scaled to historical values (i.e. shown is the difference between historic and modern values, divided by the historic). Circled points are two outliers where gains in variation are attributed to immigration and limited historical samples (*Atlapetes pallidiceps,* Hartmann et al., 2014; *Capreolus capreolus,* Wang et al., 2002)

The magnitude of change in expected heterozygosity was significantly associated with the age of the oldest sample in a study (*ê* = −0.0011 ± 0.0005 *SE, t *= −2.307, GLMM) and whether the populations occurred on an island or mainland (*ê* = 0.1908 ± 0.0671 *SE, t *= −2.846, GLMM). The final model contained only these two explanatory variables and taxonomic class as a random factor. All other variables in the model were nonsignificant, including the *IUCN Red List of Threatened Species* status (Endangered: *ê* = −0.0573 ± 0.1141 *SE*, *t *= −0.502; Vulnerable: *ê* = −0.0513 ± 0.1197 *SE, t *= −0.429; Near Threatened: *ê* = −0.0227 ± 0.1194 *SE*, *t *= −0.190; Least Concern: *ê* = −0.1036 ± 0.1086 *SE, t *= −0.945; Invasive: *ê* = −0.0899 ± 0.148 *SE*, *t *= −0.607, GLMM), bottleneck status (*ê* = 0.0195 ± 0.0569 *SE*, *t* = 0.343, GLMM) and number of generations a study spanned (*ê* = 0.0004 ± 0.0014 *SE*, *t* = 0.266, GLMM). There was a negative relationship between the total expected heterozygosity lost and age of the oldest sample (Figure 1), meaning that studies from closer to the industrial revolution showed significantly greater loss of expected heterozygosity. Island species also showed a significantly larger loss of expected heterozygosity, declining by an average of 27.6% (±28.1% *SD*) relative to an average loss of 2.1% (±15.8 *SD*) in mainland populations.

**Figure 2:**
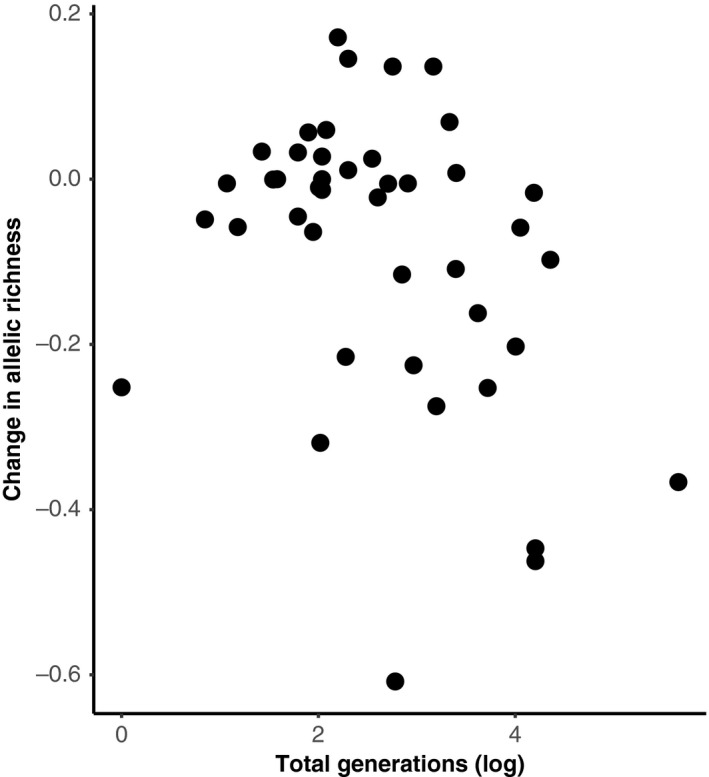
Proportional change in allelic diversity as measured by allelic richness. Values are scaled to historic levels (i.e. shown is the difference between historic and modern values, divided by the historic). The total generations have been log‐transformed to help with visualization. The untransformed generation numbers span from 1 to 300

The analysis of allelic diversity (measured by allelic richness) yielded similar results to those for expected heterozygosity. No significant effect was found for *IUCN Red List of Threatened Species* status (Endangered: *ê* = −0.1752 ± 0.0866 *SE*, *t *= −2.023; Vulnerable: *ê* = −0.0351 ± 0.0855 *SE, t *= −0.410; Near Threatened: *ê* = −0.1075 ± 0.0832 *SE*, *t *= −1.291; Least Concern: *ê *= −0.1814 ± 0.0824 *SE*, *t *= −2.201; Invasive: *ê *= −0.1975 ± 0.1038 *SE, t *= −1.903, GLMM) or bottleneck status (*ê* = 0.0007 ± 0.0044 *SE*, *t = *0.161, GLMM). In contrast to expected heterozygosity, there was also no significant effect of the age of the oldest sample (*ê *= −3^e−05^ ±7^e−05^ *SE*, *t *= −0.436, GLMM). Instead, the number of generations a study spanned had a significant association with loss of allelic diversity (*ê *= −0.001 ± 0.0004 *SE, t *= −2.307, GLMM). However, the trends across the two metrics were similar, and studies spanning more generations showed greater loss of allelic diversity (Figure 2). Island species (30.9% ±26.1 *SD*) also showed substantially greater loss of allelic diversity relative to those on a mainland (4.6% ±12.3 *SD*) (*ê *= −0.1908 ± 0.0671 *SE*, *t *= −2.846). Finally, we found that deviations from Hardy–Weinberg equilibrium proportions were not more common in either time point (χ^2^ **= 1.4104, *df* = 1, *p* = 0.235, chi‐squared test).

## 4 DISCUSSION

### 4.1 Global trends in genetic variation

We identified a significant loss in allelic diversity (6.5%) and expected heterozygosity (5.4%) of modern populations based on data from a comprehensive literature review. The overall trends of decline in expected heterozygosity and allelic diversity are consistent with previous single time‐point studies showing a putative global decline in genetic variation (DiBattista, 2008; Miraldo et al., 2016). However, the loss of allelic diversity identified here (6.5%) is smaller in magnitude than a difference previously reported between fragmented and unfragmented natural populations (29%) (DiBattista, 2008). The discrepancy between the two measures can be attributed to the difference in study designs: here, we considered all cross‐generational studies, even those where environmental conditions remained essentially constant, whereas the previous analysis exclusively examined single time‐point studies and only compared populations with a known environmental difference. The decline in genetic variation identified in this study is also less severe than many estimates of ongoing population declines. Wild vertebrate populations, for example, are estimated to have declined by 58% since 1970, at a rate of 2% per year (World Wildlife Fund, 2016), while common farmland bird populations have declined by 49% (see Pereira, Navarro, and Martins (2012) figure 4b for a clear summary across different metrics). We observed a baseline decline of ~6% in variation across an average of 110 years. This equates to a decline of ~0.05% per year. The slower loss of genetic variation relative to population size is anticipated because loss of genetic variation is broadly linked to genetically effective population size and not census size (Allendorf et al., 2013). It should be noted that this annual decline of ~0.05% should only be used comparatively because, as discussed below, loss of genetic variation is unlikely to occur evenly across time.

In contrast to the other metrics, little to no change was identified in observed heterozygosity. The unequal trends in expected and observed heterozygosity cannot be attributed to either a failure to identify alleles or increased error in historical samples, because deviations from Hardy–Weinberg equilibrium proportions were not more likely in the historic time points. Instead, the mismatch in trends is likely fuelled by an excess of observed heterozygotes in modern populations. This can arise in small or declining populations because the strong genetic drift they experience not only leads to unequal allele frequencies across populations, but can lead to unequal frequencies across the sexes (Rasmussen, 1979; Robertson, 1965). As a result of the frequency mismatch across the sexes, the observed heterozygosity in offspring cohorts will often be greater than that expected under Hardy–Weinberg equilibrium, potentially leading to the trends observed. Many historical time points were also represented by a small number of samples. While expected heterozygosity and measures of allelic diversity (notably allelic richness) are robust to small sample sizes, observed heterozygosity will be underestimated in such scenarios (Pruett & Winker, 2008). This may have lead to underestimated historical value of observed heterozygosity, preventing us from identifying any change in heterozygosity.

The studies that met our criteria were taxonomically biased. For instance, commercially important fish from the class *Actinopterygii* comprised 31% of our studies. Unsurprisingly, mammals and birds were also well represented, encompassing 14% and 24% of studies, respectively. Worryingly, few studies on insects (*n* = 12), amphibians (*n* = 3) or reptiles (*n* = 2) were found. Plant‐based studies were also not in this data set because they often failed to meet our inclusion criteria. A strong taxonomic skew towards commercially important fish, where stocking has been used over the last century, could be biasing the observed trends in genetic diversity and decreasing the loss we observe. Thus, we also compared variation trends averaged across taxonomic classes. The previously identified trends remained reasonably stable: expected heterozygosity declined by an average of 3.8% (significantly) and allelic diversity declined by 3.7% (nonsignificantly). We recommend that the taxonomic gaps be addressed, given the importance of understanding diversity trends in different taxa, for instance, in crop pollinators and nonagricultural plants.

### 4.2 Factors affecting loss of genetic variation

An approximate decline of 5.4%–6.5% in genetic variation represents a global average, and much larger declines could be occurring in some species, geographical regions or time periods. We therefore examined what factors were correlated with the loss of genetic variation using a general linear mixed‐effects model with taxonomic class as a random variable. The loss of neither allelic diversity nor expected heterozygosity was related to species’ *IUCN Red List of Threatened Species* status (International Union for Conservation of Nature, 2018). Each category of *IUCN Red List of Threatened Species* was well represented across the studies. For example, 37% of studies in our data set were on species of *Least Concern*, indicating that a decline in genetic diversity is occurring even for species of low conservation concern and that the trend of decline is not due to a bias towards *Critically Endangered* or *Endangered* species (21% of the data set). Recent research has shown that there is similarly little correlation between *Red List* ranking and contemporary genome‐wide heterozygosity, where historical bottlenecks and species‐specific life history traits were speculated to be more important (Díez‐del‐Molino et al., 2018). However, in our analysis bottleneck status also had a nonsignificant effect on the magnitude of variation lost, though metrics of genetic variation were lower in bottlenecked species. Due to the opportunistic nature of historical samples, many studies on bottlenecked populations may have been unable to sample before the declines began. If the historical baseline for genetic variation metrics was taken during or after a bottleneck, the largest effects on variation may have already occurred, preventing us from observing an effect.

The change in expected heterozygosity was significantly correlated with the date of the oldest sample in a study. Studies using archival samples from close to the end of the industrial revolution often showed a much greater decline relative to those with time series starting in, or after, the 1950s (Figure 1). The number of generations between time points was also correlated with the total loss of allelic diversity, with studies spanning more generations typically showing a greater decline (Figure 2). Habitat fragmentation and agricultural transformation intensified dramatically after the industrial revolution (Ellis et al., 2010). Consequently, many population declines may have either begun in the 1800s or intensified at this point. Estimates of variation for earlier historical time points, particularly those close to the industrial revolution, are therefore likely predecline or at least more representative of historical levels. Estimates are likely mid‐decline for historical time points in the 1950s, where rare alleles in particular may have already been lost. These effects are likely driving the trend of reduced genetic diversity loss with younger historical samples (Figure 1). Multi‐time point comparisons of the same populations, ideally using ancient DNA to obtain estimates from before the 1700s, are now needed to confirm when declines in genetic variation began.

Postindustrial revolution human disturbances, such as land use change and habitat fragmentation, are not distributed equally across the globe and are particularly severe within western Europe and South‐East Asia (Ellis et al., 2010). Furthermore, population declines since the 1970s are 30% higher in Neotropical regions relative to the global average (World Wildlife Fund, 2018). Regions of extreme change are likely suffering more severe variation loss, and it is essential to identify such locations. Unfortunately, the availability of temporal studies meeting our criteria was globally unequal, making a fine‐scale investigation into geographical trends impossible. Over 62.6% (*n* = 62) of species in the data set were from North America and Europe. This regional bias is similar to, though more extreme than, that previously found for mitochondrial haplotype diversity estimates (Miraldo et al., 2016). This is because of the rarity of historical samples, as well as the cost and specialist knowledge needed to sequence them (Wandeler et al., 2007). Though regional comparisons were impossible, we were able to compare trends in variation loss between island and continental mainland populations. Loss of genetic variation was substantially, and significantly, greater on islands relative to mainland populations. Island species lost 27.6% and 30.9% of expected heterozygosity and allelic diversity (respectively), relative to only 2.1% and 4.6% in mainland species. This matches with our expectations because island species often consist of small and isolated populations and, as a result, are more vulnerable to extreme population declines (Purvis, Gittleman, Cowlishaw, & Mace, 2000). Furthermore, human impacts measured by population density, land transformation, transport infrastructure, invasive species and electrical power infrastructure are higher on islands (Purvis et al., 2000; Sanderson et al., 2002). Together, these human influences are likely fuelling higher rates of fragmentation, population declines and genetic drift that are ultimately leading to the higher loss in expected heterozygosity on islands. Island species have previously been shown to harbour less genetic variation on average than mainland species due to founder effects and small population size (Frankham, 1997; Stuessy, Takayama, López‐Sepúlveda, & Crawford, 2014), but to our knowledge, a trend of elevated variation loss has not been previously shown. Islands are hot spots of endemism, and this severe genetic decline will disproportionally elevate the risk of global species loss (Kier et al., 2009), exacerbating the global biodiversity extinction crisis if left unmitigated.

Historical samples are known to have a higher rate of genotyping error and smaller sample sizes, both of which can decrease estimates of heterozygosity and allelic diversity (Wandeler et al., 2007). Within this study, we found no differences in probability of deviation from Hardy–Weinberg equilibrium proportions between time points, suggesting that genotyping errors were not substantially more common in historic time points. We also found that sample sizes were fairly large across both time points; thus, the trends observed here are not likely to be driven by consistently poor historical baseline estimates within data sets. A notable exception to these findings is an extreme outlier in Figure 1, where a gain in diversity was attributed to a poor historical baseline in the original study (*Atlapetes pallidiceps,* Hartmann, Schaefer, & Segelbacher, 2014). A second outlier is also present, but this was attributed by the original authors to immigration introducing new genetic variation during the study (*Capreolus capreolus,* Wang, Lang, & Schreiber, 2002). The remaining more minor variation increases are often seen in fish and are likely due to stocking efforts (where populations are supplemented with captive‐reared individuals).

An estimated average loss in genetic variation of 5.4%–6.5% may seem surprisingly low. However, our requirement for modern samples (to compare diversity change through time) forced the exclusion of extinct species. Consequently, the species that have undergone the most severe population declines are not in this analysis. Conservative estimates suggest extinction rates are now 100 times the historical baseline rate (Ceballos et al., 2015). Hence, the true total loss of genetic variation is likely to be much greater than estimated, and a ~ 6% decline should be viewed as conservative. Importantly, our study highlights that it is essential to address the taxonomic and geographical gaps in temporal studies and confirm these trends. To ensure cross‐compatibility, future studies should ensure that the classical metrics of genetic variation (expected and observed heterozygosity, allelic diversity) continue to be reported, alongside generation times and population size estimates.

## CONFLICT OF INTEREST

None declared.

## AUTHOR CONTRIBUTIONS

DML conducted the literature and statistical analysis, and wrote the paper. AH and EVD commented on the analyses and text. VF mentored the work and commented on the analyses and text. All authors contributed to the original idea.

## Data Availability

Data underlying the study, including a summary table of studies used and diversity statistics reported, are available in Dryad: https://doi.org/10.5061/dryad.8c4c359 (Leigh, Hendry, Vázquez‐Domínguez, & Friesen, 2019).
